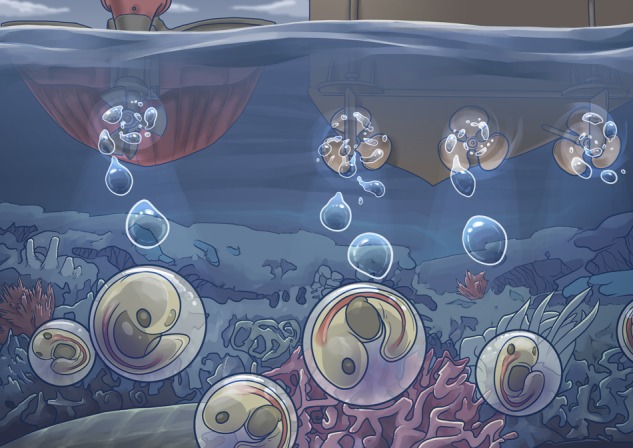# Sound the alarm: motorboat disturbance to fish embryos depends on engine type

**DOI:** 10.1093/conphys/coy064

**Published:** 2018-12-01

**Authors:** Alexander G Little

**Affiliations:** Department of Ecology, Evolution, and Marine Biology, University of California, Santa Barbara, CA 93106, USA

When asked to consider aquatic pollution, we may first think of rubbish. Noise from boat engines may not immediately come to mind. But, humans have been altering the world’s natural acoustic background with increasing intensity since the Industrial Revolution. The 20th century invention of the marine engine, coupled with a recent spike in ecotourism to remote destinations, means many aquatic species have been hit hard by punctuated increases in noise without time to adapt. A recent study from Sofia Jain-Schlaepfer and her colleagues (2018) from James Cook University in Australia and Exeter University in the UK found noise from boat engines represents a dangerous stressor for developing fish embryos. Interestingly however, not all boat engines are created equal.

In the human world, local bylaws restrict noise from raging house parties, construction equipment and airport traffic. But, under the sea, there is no such legislature to protect fish and other animals in their marine homes. Even though sound travels faster and with more energy underwater, there are not enough data to understand its impact on marine species. This is why Jain-Schlaepfer and her colleagues decided to test the effects of noise from boat engines on the early life stages of the staghorn damselfish, a coral reef dweller that lays its eggs on dead coral. Because embryo survival depends on growing big quickly, this is a key life stage to examine, as it may also be the most vulnerable to stress.

The team also wanted to know if all boat engines are created equal. In other words, are the impacts for two-stroke comparable to four-stroke outboard engines? This line of questioning is especially relevant because restricting engine properties may represent a more viable mitigation strategy for aquatic noise pollution than restricting overall boat traffic.

So, how did the team do these experiments? They drove boats, of course!

Using seven aluminium-hulled 5m long boats (4 with 30 hp Suzuki two-stroke outboard engines and three with 30 hp four-stroke outboard engines), they drove near the unsuspecting embryos at speeds of 0–35 km/h.

The team discovered that engine noise significantly increased the fish embryos’ heart rates, which likely represents a whole-animal stress response. Importantly, however, the team found that noise from two-stroke engines elevated heart rate more than twice that of four-stroke engines. Stress responses can benefit an organism when a threat is real. But, if an organism mounts a stress response to a ‘perceived threat’ that poses no real danger, it can compromise survival by redirecting already limited energy supplies away from growth and development. Indeed, more data are needed to understand just how detrimental boat noise is to embryo survival and population health. But, studies on closely related species are already suggesting that noisy environments result in smaller larval body sizes and therefore lower survival.

Noise pollution from boat engines represents a prevailing human-induced stressor for many aquatic species. Because not all boat engines are created equal, legislature to target specific engine properties likely represents the most realistic alternative—for now—to reducing overall boat traffic.

Illustration by Erin Walsh; Email: ewalsh.sci@gmail.com

**Figure:**